# Pan-cancer genetic analysis of cuproptosis and copper metabolism-related gene set

**DOI:** 10.3389/fonc.2022.952290

**Published:** 2022-10-06

**Authors:** Hengrui Liu, Tao Tang

**Affiliations:** ^1^ Department of Molecular Diagnostics, Sun Yat-Sen University Cancer Center, State Key Laboratory of Oncology in South China; Collaborative Innovation Center for Cancer Medicine, Guangzhou, China; ^2^ Yinuo Biomedical Co., Ltd, Tianjin, China

**Keywords:** cuproptosis, cancers, genes, clinical, immune

## Abstract

**Background:**

A recent paper has revealed a novel cell death pathway, cuproptosis, a programmed cell death based on copper. This study aimed to evaluate the pan-cancer genomics and clinical association of cuproptosis and copper metabolism-related cell death genes, including SLC25A3, SLC25A37, SLC31A1, FDX1, DLAT, LIAS, ATP7A, ATP7B, COX17, SCO1, SCO2, COX11, and COX19.

**Methods:**

By mining multi-omics profiling data, we performed a comprehensive and systematic characterization of cuproptosis genes across more than 9,000 samples of over 30 types of cancer.

**Results:**

ATP7B and ATP7A were the two most frequently mutated copper cell death genes in cancer. UCEC and SKCM were the two cancer types that have the highest mutation rates while the mutation of LIAS was associated with worse survival of BRCA. Brain cancer was potentially affected by copper cell death because of the difference in copper cell death gene expression among subtypes and stages. On the contrary, KIRC might have a lower cuproptosis activity because of the decrease in copper cell death gene expression. In lung cancer and kidney cancer, most of the cancer–noncancer expression patterns of copper cell death genes were consistent between mRNA and protein levels. Some of the cuproptosis gene expression was associated with the survival of LGG, KIRC, and ACC. The top five expression-copy numbers correlating cancer types were BRCA, OV, LUSC, HNSC, BLCA, and LUAD. Generally, the copy number variations of these genes in KIRC, UCEC, and LGG were associated with survival. The expression of DLAT, LIAS, and ATP7B was negatively correlated with the methylation in most of the cancer types. The copper cell death genes regulating miRNA and pathway regulation networks were constructed. The copper cell death genes were correlated with immune cell infiltration levels of multiple immune cells. These genes were correlated with the sensitivity of cancer cells to multiple drugs.

**Conclusion:**

Copper cell death genes are potentially involved in many cancer types and can be developed as candidates for cancer diagnosis, prognosis, and therapeutic biomarkers.

## Introduction

Much as the COVID-19 pandemic has impeded and delayed the diagnosis of cancers in many countries and decreased cancer new cases in the short-term, a reasonably expected rise in late-stage cancer cases and higher cancer-related mortality will occur in the next few years (1). Cancer is one of the most concerning public health issues in the world; for example, cancer accounted for 1,918,030 new cancer cases and 609,360 cancer deaths in the United States in 2022 according to the American Cancer Society (2). Therefore, more cancer investigations in the improvement of early detection and treatment would benefit the reduction in cancer death.

The fast-growing studies of ferroptosis, a relatively novel type of programmed cell death, have boosted a perspective on its usage in cancer diagnosis, prognosis, and therapeutics (3–5). Ferroptosis also raised the interest of researchers in metal-based programmed cell deaths. A recent *Science* paper (6) has revealed another metal-based programmed cell death, the cuproptosis, a programmed cell death based on copper. As an essential element for almost all organisms, copper served as a co-factor involved in the activities of many critical enzymes across the whole animal kingdom (7). However, in healthy cells, the intake and output of copper are strictly regulated to maintain a low homeostatic level of intracellular copper (8, 9). The accumulation of copper in cells leads to a detrimental or even dead fate of cells (8, 9). Copper concentrations have been found to affect many types of cancers, such as breast cancer (10), head and neck cancer (11), and endometrial cancer (12). Researchers in the field have suggested copper metabolism as a unique vulnerability for cancers (13) and copper-involving cancer therapies have been reported in many studies (14).

A novel cuproptosis pathway has been reported recently (6), which described a programmed cell death pathway where the copper directly binds to lipoylated components of the tricarboxylic acid (TCA) cycle and leads to the aggregation of lipoylated protein and the loss of downstream iron–sulfur cluster proteins. These biological processes result in proteotoxic stress, thereby inducing cell death. The cuproptosis study (6) explained the previous copper homeostatic mechanisms (15, 16) and specified the core molecules in copper-based cell death. This recent paper (6) attracted the attention of a large number of cancer researchers because cuproptosis may reveal a novel cell death pathway shared across all cancer types. Therefore, the study of the potential value of cuproptosis-related genes in cancer diagnosis, prognosis, and therapies is of great significance.

Cancer databases, such as The Cancer Genome Atlas (TCGA) (17), provide gene alteration, gene expression, and clinical survival data on different cancer types, facilitating pan-cancer studies for the identification and understanding of biomarkers or therapeutic targets for cancer treatments (18–20). This study focused on 13 core genes in copper metabolism-related cell death, including SLC25A3, SLC25A37, SLC31A1, FDX1, DLAT, LIAS, ATP7A, ATP7B, COX17, SCO1, SCO2, COX11, and COX19. The copper-related functions of these genes are summarized in **Table 1**. ATP7A and ATP7B (ATPase Copper Transporting) are copper efflux transporters (24, 29) while SLC31A1 (Solute Carrier Family 31 Member 1) is a copper importer (30). The balance of the copper transporters largely determined the homeostasis of intracellular copper concentrations. The other three focused molecules, FDX1 (ferredoxin 1), DLAT (dihydrolipoamide S-acetyltransferase), and LIAS (lipoic acid synthetase), are essential genes for cuproptosis (6). Therefore, SLC31A1, FDX1, DLAT, and LIAS are pro-cuproptosis genes while ATP7A and ATP7B are anti-cuproptosis genes. SLC25A3 and SLC25A37 encode two critical mitochondrial copper transporters (21, 22), which potentially regulate cuproptosis in cancers. In addition, we are also interested in the major intermembrane space chaperones COX17, SCO1, SCO2, COX11, and COX19 (25, 26) (27, 28). COX19 and SCO1 have been implicated in a signaling cascade that controls cellular copper (31) and therefore may be important contributors to copper cell death.

**Table 1:** Summary of copper metabolism-related cell death.

Gene symbol	Full name	Encoding protein	Function in copper metabolism	References
SLC25A3	Solute Carrier Family 25 Member 3	Phosphate carrier protein (PCP)	Mitochondrial copper transporter	(21)
SLC25A37	Solute Carrier Family 25 Member 37	Mitoferrin1 (Mfrn1)	Mitochondrial copper transporter	(22)
SLC31A1	Solute Carrier Family 31 Member 1	High-affinity copper uptake protein 1 (CTR1)	Copper transporter in the cell membrane: A homotrimer affects the uptake of dietary copper	(23)
FDX1	Ferredoxin 1	Ferredoxin 1	Transfers electrons from NADPH through ferredoxin reductase to mitochondrial cytochrome	(15)
DLAT	Dihydrolipoamide S-Acetyltransferase	Dihydrolipoamide S-Acetyltransferase	One of the key proteins in multi-enzyme pyruvate dehydrogenase complex (PDC), catalyzing the conversion of pyruvate to acetyl coenzyme A, essential molecules for cuproptosis	(6)
LIAS	Lipoic Acid Synthetase	Lipoic Acid Synthetase	An iron–sulfur enzyme catalyzes the final step in the *de novo* pathway for the biosynthesis of lipoic acid, a potent antioxidant, an essential molecule for cuproptosis	(6)
ATP7A	ATPase Copper Transporting Alpha	ATPase Copper Transporting Alpha	Membrane copper transporter	(24)
ATP7B	ATPase Copper Transporting Beta	ATPase Copper Transporting Beta	Membrane copper transporter	(24)
COX17	Cytochrome C Oxidase Copper Chaperone	Cytochrome C Oxidase Copper Chaperone	Functions importantly in copper metalation of cytochrome c oxidase and integral mitochondrial architecture	(25)
SCO1	Synthesis of Cytochrome C Oxidase 1	Synthesis of Cytochrome C Oxidase 1	Functions in the assembly of cytochrome c oxidase (COX) and cellular copper homeostasis	(26)
SCO2	Synthesis of Cytochrome C Oxidase 2	Synthesis of Cytochrome C Oxidase 2	Functions in the assembly of cytochrome c oxidase (COX) and cellular copper homeostasis	(26)
COX11	Cytochrome C Oxidase Copper Chaperone COX11	Cytochrome C Oxidase Copper Chaperone COX11	A mitochondrial copper chaperone	(27)
COX19	Cytochrome C Oxidase Assembly Factor	Cytochrome C Oxidase Assembly Factor	A mitochondrial copper chaperone	(28)

This study aimed to provide a comprehensive pan-cancer genomics and clinical association profile of these copper cell death genes for future reference. We think, with the rise of cuproptosis cancer research, that these in-time profiles will provide a genetic overview and useful information for future studies on the role of copper metabolism-related cell death in cancers.

## Methods

### Data acquisitions

The expression data with clinical information were downloaded from The Cancer Genome Atlas (TCGA) (17). The mutation or variant data were obtained from the TCGA PanCancer Atlas Studies and UniProt. Single-nucleotide variant (SNV) and copy number variant (CNV) data were from NCI Genomic Data Commons (TCGA). Methylation data were downloaded from TCGA. The reverse-phase protein array (RPPA) data were downloaded from The Cancer Proteome Atlas (TCPA). The miRNA regulation data of melatonergic regulators were collected from databases including experimentally verified (scientific papers, TarBase, miRTarBase, and mir2disease) data, and targetscan and miRanda predicted data. The immune therapy response and survival data were accessed from the Tumor Immune Dysfunction and Exclusion (TIDE) (32). The gene–drug sensitivity data were downloaded from the Drug Sensitivity in Cancer (GDSC) database and the Cancer Therapeutics Response Portal (CTRP).

### Gene alterations and expression analysis

All the expression and methylation analyses and plotting were implemented by R foundation for statistical computing (2020) version 4.0.3 and ggplot2 (v3.3.2) or accessed from the website of the data source. SNV plots were generated by the maftools (24). CNV data were processed with GISTICS2.0 (30).

### Immunohistochemistry staining

Representative immunohistochemistry staining images of copper metabolism-related cell death proteins in cancer and noncancer tissues were accessed from the Human Protein Atlas (HPA) (33). The sample details and the general pathological annotations were provided by the HPA.

### Clinical association analysis

The mRNA expression and clinical survival data were analyzed. The one-class logistic regression (OCLR) machine learning algorithm (34) was used to calculate the stemness index based on mRNA expression (mRNAsi) for the evaluation of stemness. The tumor mutational burden (TMB) (35) and microsatellite instability (MSI) (36) were used to evaluate the mutation levels of samples.

### Pathway activity analysis

Reverse-phase protein array (RPPA) data from the TCPA database were used to calculate pathway scores for 7,876 samples. Ten cancer-related pathways included tuberous sclerosis 1 protein (TSC)/mechanistic target of rapamycin (mTOR), receptor tyrosine kinase (RTK), phosphoinositide 3-kinases (PI3K)/protein kinase B (AKT), RAS/mitogen-activated protein kinase (MAPK), hormone estrogen receptor (ER), hormone androgen receptor (AR), epithelial–mesenchymal transition (EMT), the DNA damage response, cell cycle, and apoptosis pathways. The pathway score is the sum of the relative protein level of all positive regulatory components minus that of negative regulatory components in a particular pathway. The pathway activity score (PAS) was estimated as in previous studies (37, 38); gene expression was divided into two groups (High and Low) by the median expression, and the difference in PAS between groups was analyzed using Student’s *t*-test where the *p*-value was adjusted by the FDR. FDR ≤ 0.05 was considered significant. When PAS (Gene A group High) > PAS (Gene A group Low), Gene A was considered to have an activating effect on this pathway; otherwise, it had an inhibitory effect on the pathway.

### MicroRNA regulation network analysis

As some of the microRNA (miRNA)–gene pairs were not recorded in the databases, only miRNA–gene pairs that have recorded data were used to calculate the expression correlation. The miRNA expression and gene expression were merged *via* the TCGA barcode. The association between paired mRNA and miRNA expression was tested based on a Pearson product-moment correlation coefficient and the t-distribution. The *p*-value was adjusted by the FDR and only significant connections were plotted. Correlations were calculated for all paired samples. Meanwhile, in consideration of the presence of positive regulators, including transcription factors, a miRNA–gene pair with a negative correlation will be considered as a potential negatively regulated pair. The network was constructed using the visNetwork R package.

### Immune association analysis

Immune cell levels in cancers were analyzed using TCGA data. The infiltrates of 24 immune cells were evaluated using the ImmuCellAI (39). GSVA scores of copper metabolism-related cell death genes were used to visualize the data. The association between immune cells’ infiltrates and gene set expression level was represented by the correlation coefficient, which was evaluated through Spearman correlation analysis. The *p*-value was adjusted by FDR.

### Drug sensitivity analysis

Drugs were screened based on their CENPA correlation with drug sensitivity with a cutoff of remarkable significance (*p* < 1e-5). The GSCALite (40) was used to evaluate the area under the dose–response curve (AUC) values for drugs and gene expression profiles of CENPA in different cancer cell lines. Drug sensitivity and gene expression profiling data of cancer cell lines in the GDSC (41) and the CTRP (32) are integrated for investigation. The expression of each gene in the gene set was performed by Spearman correlation analysis with the small molecule/drug sensitivity.

### Statistical analysis

All statistical analyses were performed using the R software v4.0.3. Correlation analysis was performed using the Spearman correlation test. A Cox proportional hazards model was used to calculate survival risk and hazard ratio (HR). The prognostic significance of every variable was estimated using Kaplan–Meier survival curves and compared using log-rank tests. Group comparisons were analyzed using *t*-test or ANOVA *t*-test. If not otherwise stated, the rank-sum test detected two sets of data, and a *p*-value <0.05 was considered statistically significant.

## Results

### Single-nucleotide variation of copper cell death genes in cancers

**Figure 1:**
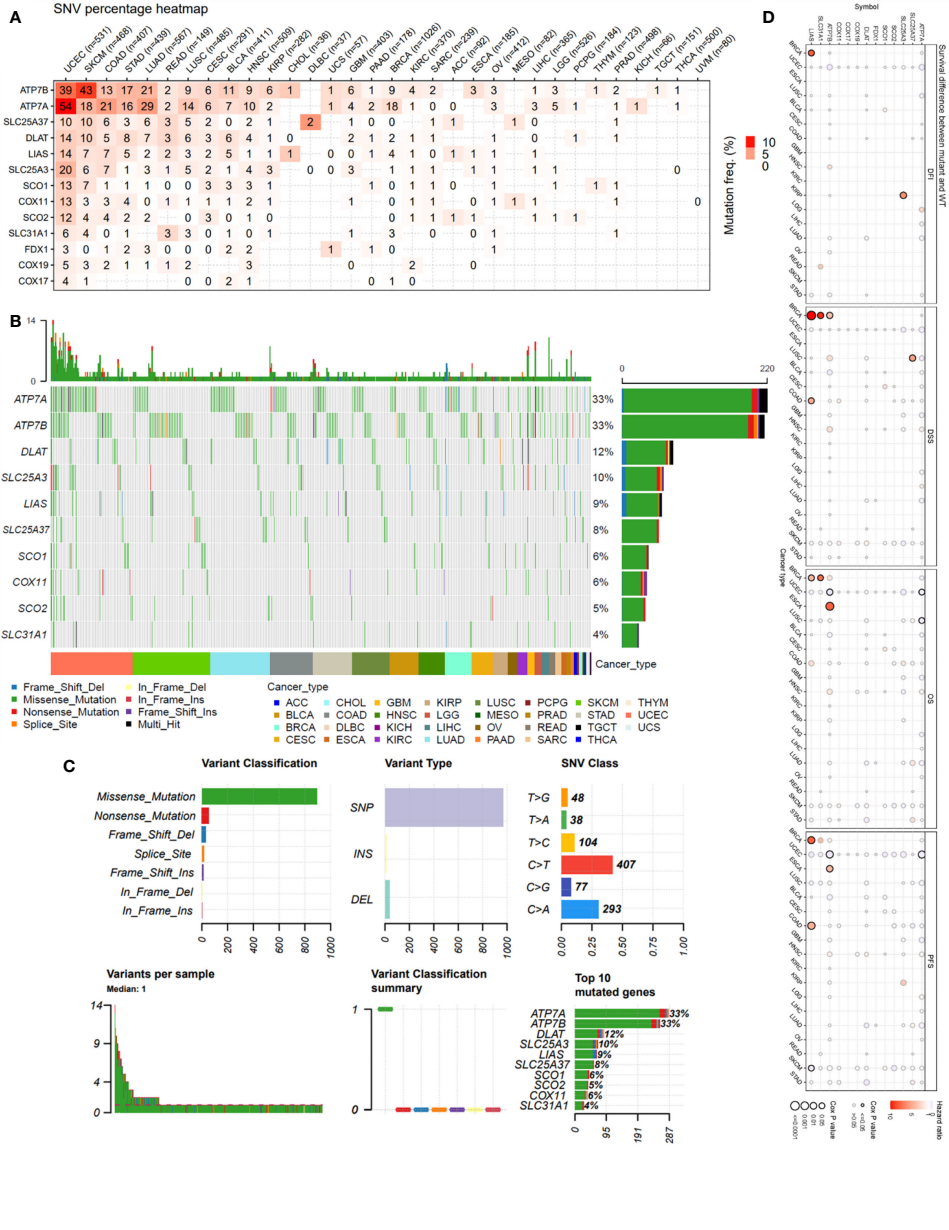
Single-nucleotide variation (SNV) analysis of copper metabolism-related cell death genes in cancers. **(A)** Heatmap of the mutation frequency. Numbers represent the number of samples that have the corresponding mutated gene for a given cancer. “0” indicates that there was no mutation in the gene coding region, blank indicates there was no mutation in any region of the gene, and color represents the mutation frequency. **(B)** The SNV landscaped plot of top 10 copper metabolism-related cell death genes in cancers. **(C)** Summarization of the SNV classes of copper metabolism-related cell death genes in cancers, including the count of each type of deleterious mutation, the count of variant types (SNP, INS, and DEL), the count of each SNV class, the count of variants in each sample (a bar indicates a sample, and the color of the bar corresponds to the color of variant classification), box plot showing the distribution of the count of each variant classification in the sample set (the color of the box corresponds to the color of variant classification), and the count and percentage of variants in the top 10 mutated genes. **(D)** Survival difference between mutant and wild-type copper metabolism-related cell death genes.

The single-nucleotide variation (SNV) analysis revealed that ATP7B and ATP7A were the two most frequently mutated copper metabolism-related cell death genes in cancer. For example, UCEC had 39 ATP7B SNV samples among 531 samples while SKCM had 43 SNV samples among 468 samples. These two cancer types were also the most mutated cancer types for the other copper cell death genes (**Figure 1A**). The SNV landscaped plot of copper metabolism-related cell death genes in cancers clearly displayed various SNV types’ distribution across cancer types (**Figure 1B**). The statistics revealed that most of the SNVs were missense-mutation. Single-nucleotide polymorphisms (SNPs) are the most common form of genetic variation. The most SNV classes were C>T and C>A (**Figure 1C**). In addition, we also analyzed and provided a profile of the association of SNV of copper cell death genes and survival. The top cancer type associated with SNV of copper cell death genes was BRCA, which had significance in the correlation between SNV of LIAS and SLC31A1 and overall survival. However, for most of the other cancer types, the SNV was not significantly associated with survival (**Figure 1D**).

### Expression of copper cell death genes in cancers

**Figure 2:**
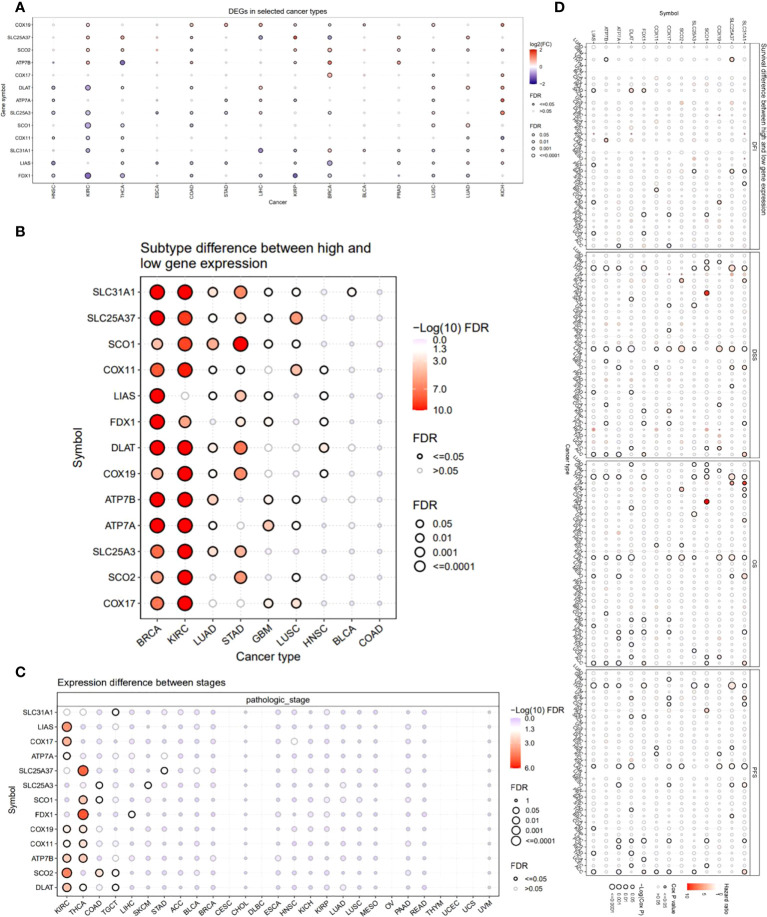
Expression and survival analysis of copper metabolism-related cell death genes. **(A)** The mRNA differences between normal samples and tumor samples. **(B)** Subtype difference between high and low gene expression samples. **(C)** Expression difference between different pathological stages. **(D)** Survival analysis of copper metabolism-related cell death genes. The dot size represents the significance of the gene’s effects on survival in each cancer type and the color represents the hazard ratio. DFI, disease-free interval; DSS, disease-specific survival; OS, overall survival; PFS, progression-free survival.

We also provided an overview of the expression profile of copper cell death genes in cancer. The comparisons of cancer and noncancer tissue expression revealed that the copper cell death genes were differentially expressed in multiple cancer types, yet the overexpression or underexpression varied from cancer type to cancer type (**Figure 2A**). For example, ATP7B was overexpressed in KIRC but under-expressed in THCA, indicating that the mechanisms of copper death in different cancer types were different. Brain cancers (GBM and LGG) were found to potentially have a higher copper metabolism-related cell death compared to normal brain tissue because their anti-cuproptosis gene ATP7B was downregulated with pro-cuproptosis genes SLC31A1, FDX1, DLAT, and LIAS upregulated in cancer tissues. On the contrary, KIRCs were found to potentially have a lower copper cell death in cancer tissue because their anti-cuproptosis gene ATP7B was upregulated with pro-copper metabolism-related cell death genes SLC31A1, FDX1, DLAT, and LIAS downregulated in cancer tissues. For LUAD and LUSC, the four pro-cuproptosis genes were overexpressed in cancer. These data strongly suggested that lung cancer has a higher copper cell death activity. To validate the expression patterns at the protein level, we accessed tissue section protein staining data of these genes. We validated the expression of the five proteins in normal vs. cancer in seven tissue types that have large sample sizes in the TCGA database with the most significance, namely, breast cancer, colon cancer, kidney cancer, lung cancer, prostate cancer, stomach cancer, and thyroid cancer. Results showed that the cancer–noncancer protein expression patterns of ATP7B, SLC31A1, DLAT, and LIAS were mostly consistent with the patterns at the mRNA level but that of FDX1 was not (**Supplementary Figure 1**). Yet, the protein expression of FDX1 in most of the samples was “low” or “very low”, indicating that the protein used for staining might not provide strong enough signals for observation. These cancer types included some cancer types we proposed to have abnormal copper cell death activity based on mRNA expression data; thus, we confirmed the observation that kidney cancer might have a lower copper cell death, while lung cancer might have a higher copper cell death activity. In the analysis of gene expression subtype differences, BRCA and KIRC were found to be the most significant cancer types (**Figure 2B**). As for the analysis of pathological stage differences, KIRC and THCA were the most significant cancer types (**Figure 2C**). In addition, we also analyzed and provided a profile of the association of copper metabolism-related cell death genes and disease-free interval, disease-specific survival, overall survival, and progression-free survival. The top cancer types with survival associated with copper cell death genes were LGG, KIRC, and ACC (**Figure 2D**). These results indicated that the regulated expression of copper cell death genes might be involved in tumorigenesis and tumor development.

### Copy number variation of copper cell death genes in cancers

**Figure 3:**
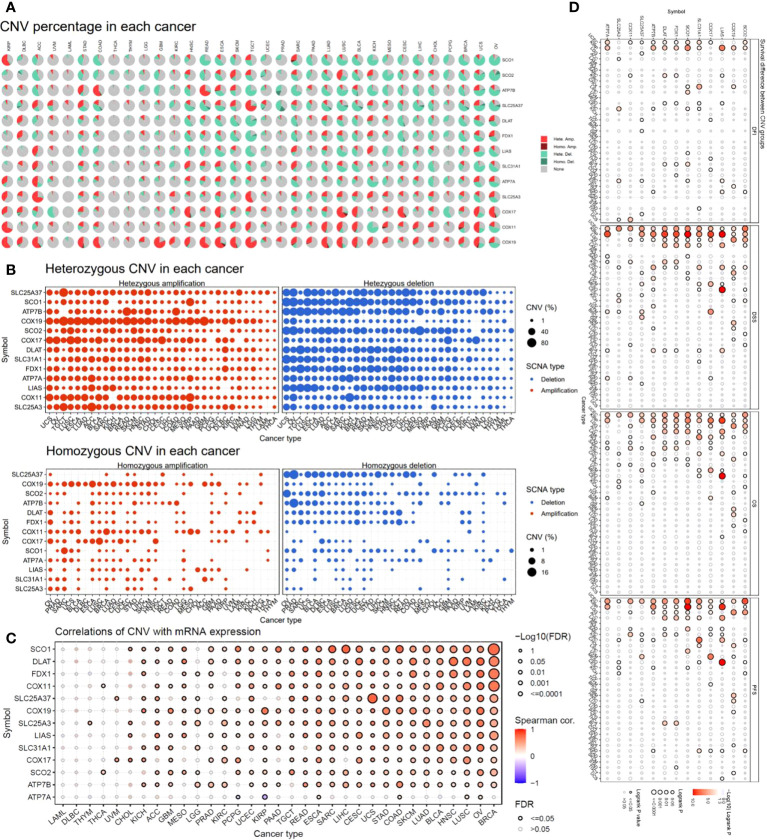
Copy number variation (CNV) analysis of copper metabolism-related cell death genes in cancers. **(A)** CNV distribution pie chart across cancers. Hete Amp, heterozygous amplification; Hete Del, heterozygous deletion; Homo Amp, homozygous amplification; Homo Del, homozygous deletion; None, no CNV. **(B)** Heterozygous CNV profile showing the percentage of heterozygous CNVs, including the percentage of amplification and deletion of heterozygous CNVs for each gene in each cancer. Only genes with >5% CNV in a given cancer are shown as a point in the figure. **(C)** The correlation of CNV and mRNA expression. **(D)** Survival difference between CNV groups.

To investigate the factors that potentially affect the expression of the copper cell death genes in cancers, we analyzed and presented the copy number variation (CNV) of these genes in cancers. The CNV analysis revealed that different cancer types had a variety of CNV patterns. The CNV pie chart distribution showed that the main CNV types were heterozygous amplification and deletion (**Figure 3A**). Thus, we further plotted the heterozygous amplification and deletion of the copper metabolism-related cell death genes in cancers for future reference (**Figure 3B**). We also calculated the correlation between CNV and expression to evaluate the effect of CNV on expression. More than half of the cancer types showed significance in correlations. The top five correlated cancer types were BRCA, OV, LUSC, HNSC, BLCA, and LUAD (**Figure 3C**). These results indicated that CNV might potentially affect the expression of the copper metabolism-related cell death genes in cancers. Interestingly, the analysis of the correlation between CNV and survival across multiple cancer types found that KIRP, UCEC, LGG, and ACC were the three cancer types where the CNV level was associated with survival (**Figure 3D**).

### Methylation of copper cell death genes in cancers

**Figure 4:**
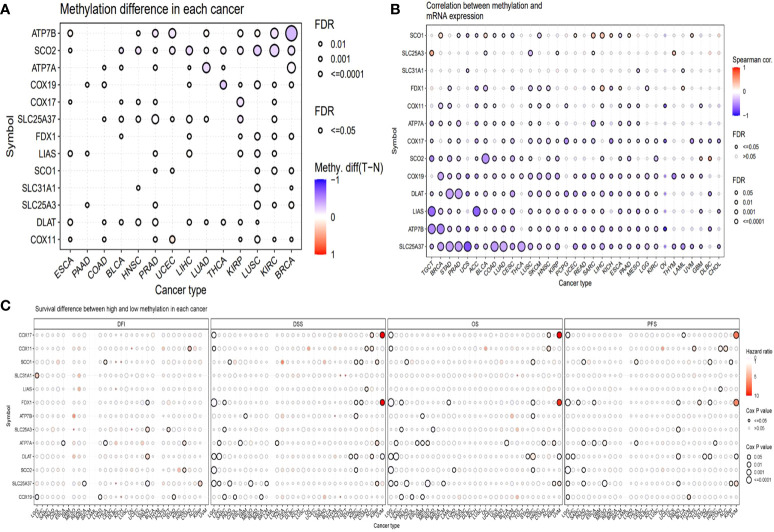
Methylation analysis of copper metabolism-related cell death genes in cancers. **(A)** Methylation difference between tumor and normal tissues. **(B)** The correlation between methylation and mRNA gene expression. **(C)** Survival difference between samples with high and low methylation of copper metabolism-related cell death genes.

Methylation is another factor that might affect the expression of these genes. Hence, we analyzed the methylation. We compared the methylation difference between cancer and normal tissues. The top three different cancer types were BRCA, KIRC, and LUSC (**Figure 4A**). The correlations between methylation and survival across multiple cancer types revealed that the expression of half of the genes showed a negative correlation with the methylation. For example, the expression of DLAT, LIAS, and ATP7B was negatively correlated with the methylation in most of the cancer types, while the expression of SLC31A and FDX1 was not remarkably correlated with the methylation in most of the cancer types (**Figure 4B**). The survival correlation analysis of methylation showed that the methylation levels were mostly not correlated with the survival of cancer in most cancer types (**Figure 4C**).

### miRNA regulation network of copper cell death genes in cancers

Another factor that potentially affects the expression of the copper metabolism-related cell death genes in cancers is the regulation of miRNA; thus, we predicted the miRNA regulation network by exploring multiple miRNA databases and analyzing the correlation between target genes and miRNAs. In the network, a miRNA and one regulator connection node represent miRNA regulation of a gene. Node size is positively correlated with the node’s degree, and edge width is defined by the absolute value of the correlation coefficient. As shown in miRNA network supplementary material, miRNAs might be involved in the regulation of the expression of the copper metabolism-related cell death core genes. For example, DLAT was regulated by 15 miRNA, where the top regulating miRNAs were hsa-miR-365a-3p, hsa-miR-664a-3p, and hsa-miR-1271-5p. LIAS was regulated by hsa-miR-452-5p and hsa-miR-1976. FDX1 was regulated by 10 miRNA and the top regulating one was hsa-miR-21-5p, which was also regulating SLC31A1. SLC31A1 was regulated by seven miRNAs, especially by hsa-miR-708-5p and hsa-let-7i-5p. ATP7B was regulated by 11 miRNAs, where the top correlated miRNAs were hsa-miR-98-5p and hsa-miR-185-5p. The network provided an overview of the potential copper metabolism-related cell death-regulating miRNAs for future studies.

### Association profile of copper cell death genes and cancer biological processes in cancers

**Figure 5:**
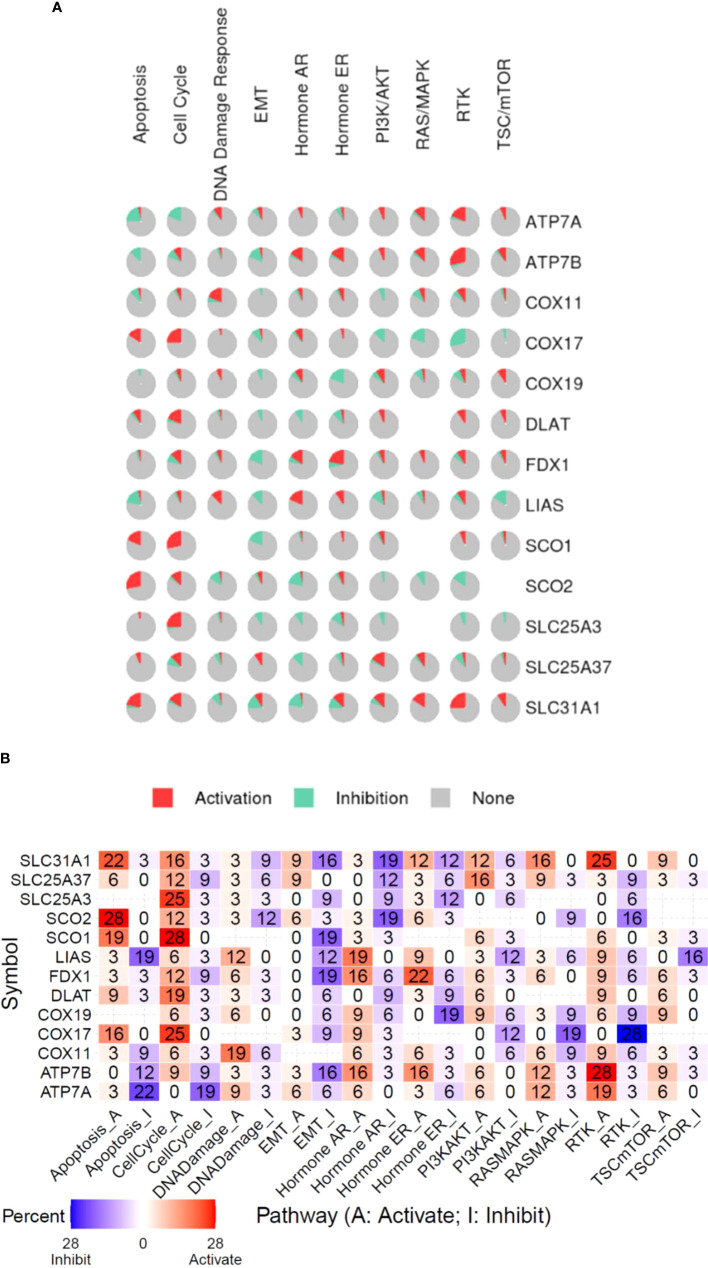
Functional pathway association of copper metabolism-related cell death genes in cancers. **(A)** The percentage pie chart of the effect of copper metabolism-related cell death genes on pathway activity. **(B)** The combined percentage of the effect of copper metabolism-related cell death genes on pathway activity.

To investigate the association between copper cell death and other cancer biological processes, we analyzed five of the copper metabolism-related cell death genes regarding the stemness, TMB, and MSI correlation in cancer. Results showed that these genes were correlated with stemness, TMB, and MSI in a few cancer types only. In the stemness analysis, STAD was the most striking cancer type, which was positively correlated with the expression of ATP7B, SLC31A1, FDX1, and DLAT. The stemness of KIRC was found to be positively correlated with the expression of FDX1. In the TMB analysis, the most correlated cancer type was DLBC, but the correlations were not striking. In the MSI analysis, only UCEC was strikingly correlated with MSI (**Supplementary Figure 2**). On the other hand, the potential effects of the copper metabolism-related cell death proteins on common cancer pathways were analyzed. The related pathways network indicated that five copper metabolism-related cell death genes were potentially involved in other cancer-related signaling pathways, including TSC/mTOR, RTK, RAS/MAPK, PI3K/AKT, hormone ER, hormone AR, EMT, DNA damage response, cell cycle, and apoptosis pathways. For example, most pathways were found to be affected by the pro-cuproptosis protein SLC31A1. All three essential cuproptosis proteins FDX1, DLAT, and LIAS were found to be potentially involved in the activation of the cell cycle pathway (**Figure 5**).

### Immune and drug sensitivity association profile of copper cell death genes in cancers

**Figure 6:**
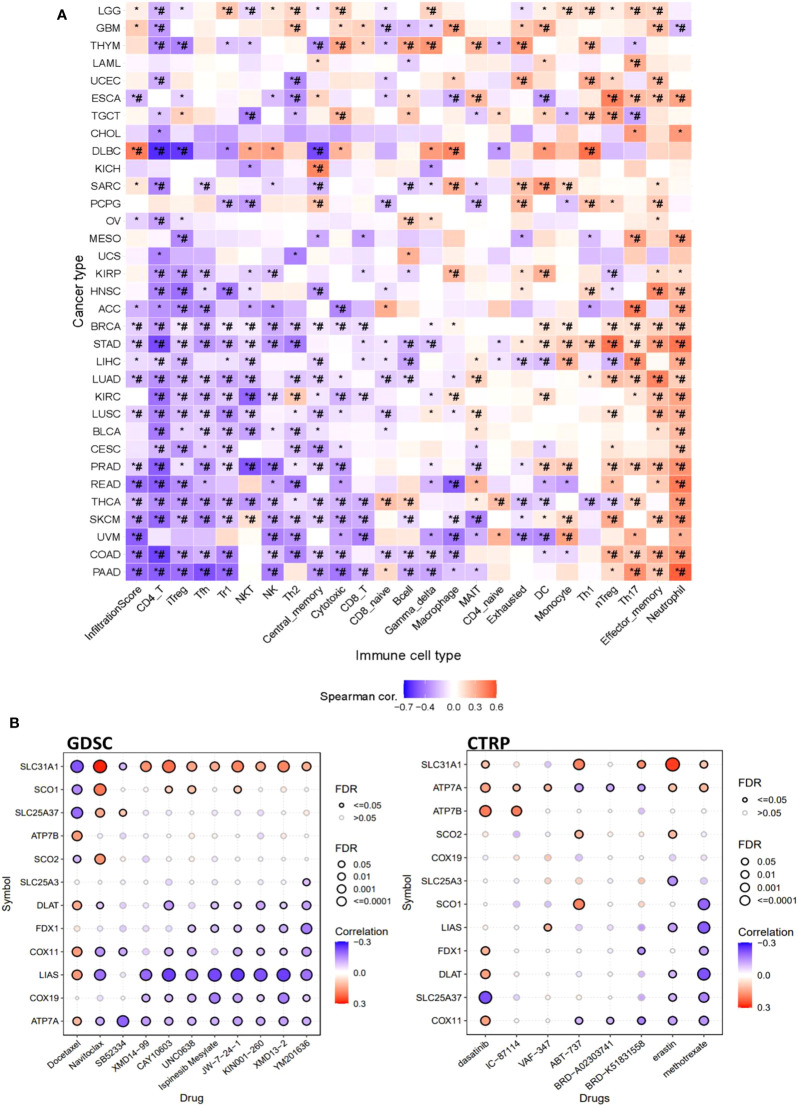
The immune and drug sensitivity analysis of copper metabolism-related cell death genes in cancers. **(A)** Correlations of the GSVA score of copper metabolism-related cell death genes and immune cell infiltration levels across cancer types. *p < 0.05;*#*p* < 0.01. **(B)** Correlation of copper metabolism-related cell death gene expression and small molecule/drug sensitivity of cancer cell lines. Left panel: GDSC data; right panel: CTRP data. Drug sensitivity and gene expression profiling data of multiple cancer cell lines in GDSC and CTRP were integrated for investigation. The expression of copper metabolism-related cell death genes was performed by Spearman correlation analysis with the small molecule/drug sensitivity (area under the IC_50_ curve).

Furthermore, this study also explored the association of copper cell death genes and tumor immune microenvironment. To facilitate the analysis, we combined the copper metabolism-related cell death genes into a gene set signature (GSVA score) for analysis of immune cell infiltration. The immune cell infiltration analysis revealed that the GSVA score was significantly correlated with the infiltration levels of multiple immune cells across multiple cancer types. In particular, the GSVA score was negatively correlated with CD4+ T and positively correlated with neutrophil cells in a majority of cancer types (**Figure 6A**). These data suggested that copper metabolism-related cell death might affect immune cell infiltration levels in cancers. Moreover, we also provide the drug sensitivity association profile of copper cell death genes in cancer cell lines using GDSC and CTRP databases. The databases provided the IC_50_ of cell lines of different cancer types to a number of compounds and also provided the expression of genes in these cell lines. We downloaded these data and analyzed the correlation between genes and the sensitivity of cell lines to these compounds. Only significant results were shown. Results showed that the copper metabolism-related cell death genes were significantly correlated with the sensitivity of cancer cells to multiple compound drugs (**Figure 6B**). We identified these compounds as potential drugs targeting copper metabolism-related cell death for future study.

## Discussion

The purpose of most cancer treatments is to reduce cancer cells by killing cancer cells or preventing cancer cell migrations. The most fundamental way to achieve the reduction of cancer cells is to induce cell death in cancer; hence, any type of cell death is of great significance to cancer researchers. Known cell death pathways, such as apoptosis, necrosis, oncosis, pyroptosis, autophagy, and ferroptosis, have been explored for their values in cancer diagnosis, prognosis, and therapeutics (3–5, 42). We suggest that the recently discovered cell death pathway, cuproptosis, will be the next hotspot in cancer cell death studies. The study of cuproptosis core genes in cancer is necessary to understand cuproptosis-related tumorigenesis and to explore the cuproptosis pathway as a potential therapeutic target for clinical cancer treatments. By mining multi-omics profiling data, we performed a comprehensive and systematic characterization of the copper cell death genes across more than 9,000 samples of over 30 types of cancer. Our results not only revealed diverse mechanisms of the regulations of copper cell death gene expression in cancer contexts but also analyzed the potential associations between copper cell death and other common cancer pathways, providing an overall picture of copper metabolism-related cell death in cancer.

FDX1 and LIAS had been validated as essential proteins for cuproptosis by knockout models (6). The decrease in copper chaperone glutathione induced cell death mediated by decreased lipoylation and the rise in DLAT oligomerization (6). The previously reported cuproptosis model suggested that DLAT, FDX1, and LIAS were three key upstream pivots in the cuproptosis pathway axis. Therefore, the alterations of these three core genes in mutation or expression, and any modulations in the mutation and expression can result in changes in the downstream cuproptosis. Many previous studies suggested that chelators (43–46) and ionophores (47–50) of copper can be used to treat cancers. These inferred the potential values of the cuproptosis pathway for the targeted therapy of cancer. A strategy for cancer treatment is to activate the cuproptosis pathway, which results in the programmed death of cancer cells. The expression differences and the differences in the expression regulation mechanisms between cancer and normal tissues provided by this study indicated the potential cancer-specific targets for target therapy activating the cuproptosis pathway. The cuproptosis was reported to be mediated by protein lipoylation (51, 52). However, to date, only a small number of protein types have been found to be lipoylated in humans, most of which are discovered in the TCA cycle, where lipoylation is essential for the function of enzymes (51, 52).

The downstream genes of the cuproptosis pathway were not investigated in this study; however, the cuproptosis-based target therapy might largely depend on the downstream elements. Thus, further study of these downstream targets will be valuable for the development of cuproptosis-based target therapy. In addition, the miRNA network analysis also provided potential therapeutic targets for cuproptosis-based therapy. The interactions were provided by the databases including some experimentally verified data and predicted data. Some of these interactions were validated by experimental evidence. For example, miR-133a was reported to downregulate ATP7B expression. The SOX9/miR-130a/CTR1 axis was reported to modulate cervical cancer cells. A study has also reported that the lncRNA XIST/miR-125a-5p/CDKN2A regulatory axis played an essential role in the development of UCEC (53). However, so far, studies in this field are still lacking. This study provided potential copper-related miRNA for future study.

Cuproptosis is a double-edged sword: genetic variation in copper homeostasis can cause lethal disorders in humans, such as Wilson’s disease and neurodegenerative disorders (54, 55). This is because cuproptosis might lead to the death of the normal cell; yet, if the cuproptosis happens in cancer, it can contribute to cancer treatment. So far, it is not sure if the cuproptosis pathway is activated or not in either immune therapy or chemotherapy. Our data suggested that the cuproptosis genes were correlated with the infiltration levels of multiple immune cells across different cancer types. These results revealed the association between cuproptosis and tumor immune microenvironments. Yet, these correlations only demonstrated the potential association; whether cuproptosis can functionally affect immune cells or whether the immune cell infiltration can induce cuproptosis will be the next scientific question to be answered. Our analysis also indicated that the cuproptosis core gene signature might be useful for the prediction of immune therapy response and survival of immune therapy patients. However, these analyses were based on retrospective data and further validations with prospective evidence are needed.

For the association between copper metabolism-related cell death and drug sensitivity, ATP7B has been reported to mediate the drug resistance of platinum in cancers, such as ovarian cancer (29) and head and neck cancer (56). SLC31A1 was also reported to be associated with drug resistance (57, 58). Yet, whether these drug resistances were associated with the copper cell death pathway remains unclear. Our analysis revealed that the expression of copper cell death genes in cancer was associated with the sensitivities of cancer cells to multiple chemicals. Multiple cancer cell lines were integrated for the calculation; thus, the analysis provided a general view that the copper cell death might potentially affect drug resistance. However, the patterns of the correlations were different, including some positive and some negative correlations. This indicated that the mechanisms of copper metabolism-related cell death in drug resistance might be different among different drugs. Hence, detailed studies to explore the mechanisms of copper metabolism-related cell death in different drug resistance are required.

Granted, all these analysis results are interesting but sometimes opposite to what we expected in their role in copper cell death. This might be accepted because so far there is no widely accepted hypothesis for why the aggregation of lipoylated proteins induces cell death in copper-related pathways; thus, it is unknown whether high or low expression of them would be more detrimental or beneficial to cell death.

## Conclusion

This study provided a pan-cancer overview of cuproptosis and copper metabolism-related cell death genes. Some cancer types aberrantly express these genes or have an abnormal level of mutation in these genes. These genes also potentially affect cancer survival and cross-react with other cancer pathways. The impact of these genes on the cancer microenvironment and drug resistance suggested their potential value in cancer therapies. Therefore, the cuproptosis and copper metabolism-related gene set can be developed as candidates for cancer diagnosis, prognosis, and therapeutic biomarkers.

## Data availability statement

The original contributions presented in the study are included in the article/**Supplementary Material**. Further inquiries can be directed to the corresponding author.

## Author contributions

All the analysis works were done by HL. HL and TT wrote the paper. TT edited the paper and supervised the project. All authors contributed to the article and approved the submitted version.

## Acknowledgments

The authors thank the support of the GSCALite (40), Dr. Jieling Weng, Weifen Chen, Zongxiong Liu, and Yaqi Yang.

## Conflict of interest

Author HL was employed by Yinuo Biomedical Co., Ltd.

The remaining author declares that the research was conducted in the absence of any commercial or financial relationships that could be construed as a potential conflict of interest.

## Publisher’s note

All claims expressed in this article are solely those of the authors and do not necessarily represent those of their affiliated organizations, or those of the publisher, the editors and the reviewers. Any product that may be evaluated in this article, or claim that may be made by its manufacturer, is not guaranteed or endorsed by the publisher.

## Glossary

**Table:**

ACC	Adrenocortical carcinoma
BLCA	Bladder urothelial carcinoma
BRCA	Breast invasive carcinoma
CESC	Cervical squamous cell carcinoma and endocervical adenocarcinoma
CHOL	Cholangiocarcinoma
COAD	Colon adenocarcinoma
DLBC	Lymphoid neoplasm diffuse large B-cell lymphoma
ESCA	Esophageal carcinoma
GBM	Glioblastoma multiforme
HNSC	Head and neck squamous cell carcinoma
KICH	Kidney chromophobe
KIRC	Kidney renal clear cell carcinoma
KIRP	Kidney renal papillary cell carcinoma
LAML	Acute myeloid leukemia
LGG	Brain lower grade glioma
LIHC	Liver hepatocellular carcinoma
LUAD	Lung adenocarcinoma
LUSC	Lung squamous cell carcinoma
MESO	Mesothelioma
OV	Ovarian serous cystadenocarcinoma
PAAD	Pancreatic adenocarcinoma
PCPG	Pheochromocytoma and paraganglioma
PRAD	Prostate adenocarcinoma
READ	Rectum adenocarcinoma
SARC	Sarcoma
SKCM	Skin cutaneous melanoma
STAD	Stomach adenocarcinoma
TGCT	Testicular germ cell tumors
THCA	Thyroid carcinoma
THYM	Thymoma
UCEC	Uterine corpus endometrial carcinoma
UCS	Uterine carcinosarcoma
UVM	Uveal melanoma
