# Supplementary material for: Pan-cancer genetic analysis of cuproptosis and copper metabolism-related gene set
Source: Front Oncol. 2022 Oct 6;12:952290. doi: 10.3389/fonc.2022.952290 (PMC9582932; doi:10.3389/fonc.2022.952290)
Supplement: Supplementary file 1 [file DataSheet_1.docx]

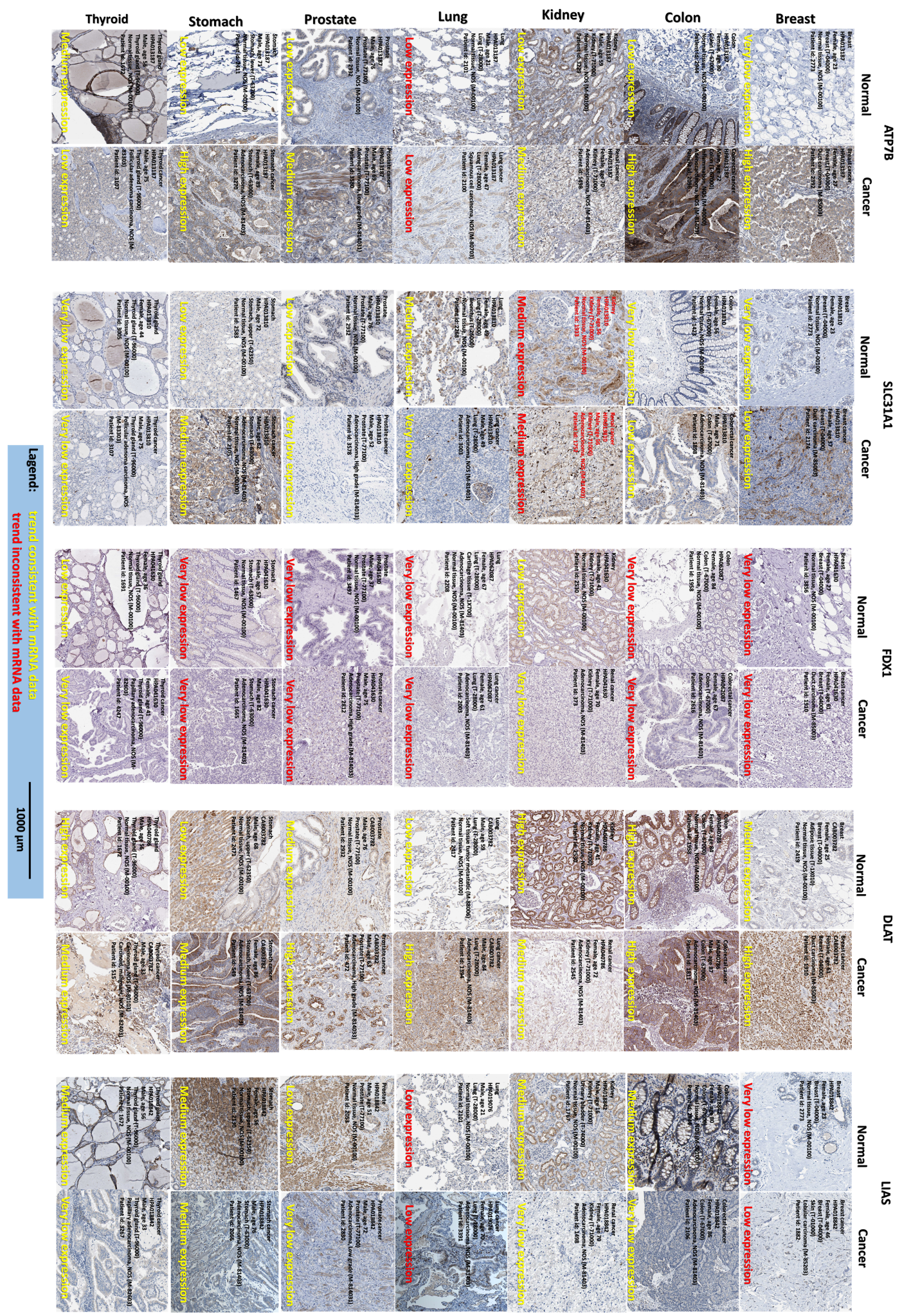


**S-Figure 1.** Representative protein staining images of cuproptosis core proteins in cancer and normal tissues. The images were accessed from the Human Protein Atlas (HPA). The sample details and the expression assessment were provided by the HPA.


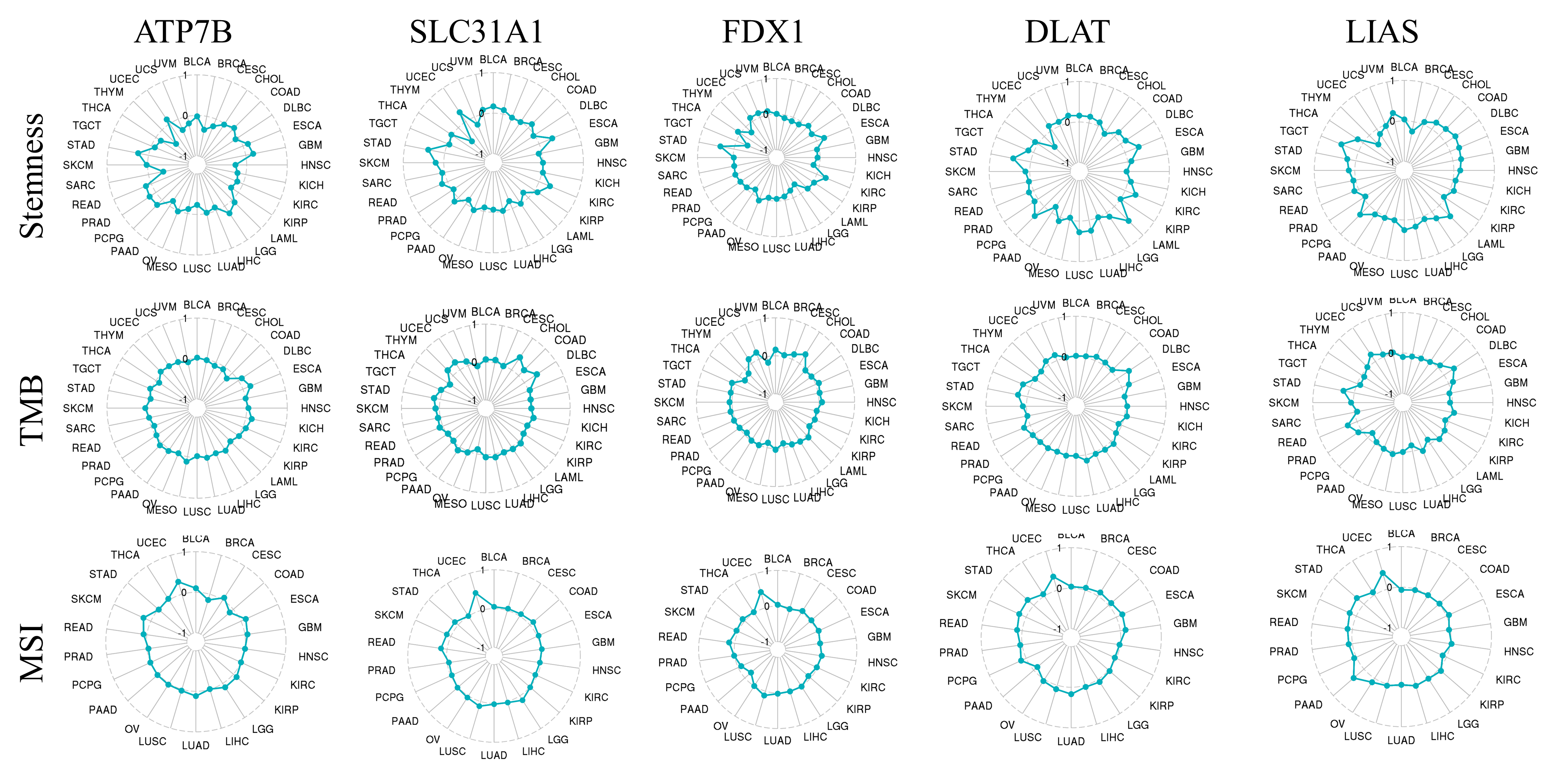


**S-Figure 2.** Representative copper metabolism-related cell death gene correlations of stemness, TMB, and MSI scores in cancer.

https://drive.google.com/uc?export=download&id=1sV-DcNDM6AKIknBQRAYe7vu0aATAgT-R

**miRNA network (supplementary material).** The miRNA network analysis. The microRNA (miRNA) network of copper metabolism-related cell death genes. A miRNA and one regulator connection node represent miRNA regulation of a gene. Node size is positively correlated with the node's degree, and edge width is defined by the absolute value of the correlation coefficient.
